# Characterization of bladder sensory neurons in the context of myelination, receptors for pain modulators, and acute responses to bladder inflammation

**DOI:** 10.3389/fnins.2013.00206

**Published:** 2013-11-07

**Authors:** Shelley L. Forrest, Peregrine B. Osborne, Janet R. Keast

**Affiliations:** ^1^Pain Management Research Institute and Kolling Institute, University of Sydney at Royal North Shore HospitalSydney, NSW, Australia; ^2^Department of Anatomy and Neuroscience, University of MelbourneMelbourne, VIC, Australia

**Keywords:** dorsal root ganglion, painful bladder syndrome, pelvic pain, visceral pain, steroid, cystitis, interstitial cystitis, inflammatory pain

## Abstract

Bladder sensation is mediated by lumbosacral dorsal root ganglion neurons and is essential for normal voiding and nociception. Numerous electrophysiological, structural, and molecular changes occur in these neurons following inflammation. Defining which neurons undergo these changes is critical for understanding the mechanism underlying bladder pain and dysfunction. Our first aim was to define the chemical classes of bladder sensory neurons that express receptors for the endogenous modulators of nociceptor sensitivity, glial cell line-derived neurotrophic factor (GDNF), the related neurotrophic factor, artemin, and estrogens. Bladder sensory neurons of adult female Sprague-Dawley rats were identified with retrograde tracer. Diverse groups of neurons express these receptors, and some neurons express receptors for both neurotrophic factors and estrogens. Lumbar and sacral sensory neurons showed some distinct differences in their expression profile. We also distinguished the chemical profile of myelinated and unmyelinated bladder sensory neurons. Our second aim was to identify bladder sensory neurons likely to be undergoing structural remodeling during inflammation. Following systemic administration of cyclophosphamide (CYP), its renal metabolite acrolein causes transient urothelial loss, exposing local afferent terminals to a toxic environment. CYP induced expression of the injury-related immediate-early gene product, activating transcription factor-3 (ATF-3), in a small population of sacral nitrergic bladder sensory neurons. In conclusion, we have defined the bladder sensory neurons that express receptors for GDNF, artemin and estrogens. Our study has also identified a sub-population of sacral sensory neurons that are likely to be undergoing structural remodeling during acute inflammation of the bladder. Together these results contribute to increased understanding of the neurons that are known to be involved in pain modulation and hyperreflexia during inflammation.

## Introduction

Visceral pain is one of the most frequent types of pain seen in the clinical setting. It is poorly localized, associated with referred pain and is resistant to many analgesics that alleviate somatic pain. Development of appropriate animal models of visceral pain is challenging, but bladder inflammation models in rodents have revealed many changes in bladder afferent pathways and their targets in the lumbosacral spinal cord that underpin nociceptor sensitization [reviewed in De Groat and Yoshimura (2009); Daly et al. (2011); Kanai (2011)]. This sensitization is critically linked to the initiation of pain and altered reflex behaviors, including increased voiding frequency and a reduction in micturition threshold, characteristics of clinical bladder inflammation.

One of the most thoroughly characterized rodent models of bladder inflammatory pain is induced by the systemic administration of cyclophosphamide (CYP), which is then metabolized to acrolein that initiates inflammation of the lower urinary tract (Cox, 1979; Maggi et al., 1992). Following either acute or chronic CYP treatment, bladder afferent neurons undergo changes in neuropeptide and ion channel expression, leading to altered excitability and connectivity, together thought to drive spontaneous activity and increased sensitivity (Nazif et al., 2007; De Groat and Yoshimura, 2009; Kanai, 2011). Defining which particular afferent neurons undergo these changes is critical to understanding the overall mechanism underlying pain and bladder dysfunction.

Primary afferent neurons that innervate the bladder are located in dorsal root ganglia (DRG). In rats these are aggregated in spinal levels L1-L2 (“lumbar”) and L6-S1 (“sacral”) (Keast and De Groat, 1992). These neurons can be identified by applying retrograde tracer to the bladder *in vivo*, a number of days prior to removal of DRGs for analysis. Using this approach, immunohistochemical studies have determined that many of these neurons synthesize neuropeptides commonly associated with sensory and nociceptive pathways, such as calcitonin gene-related peptide (CGRP) and substance P (Vizzard, 2001; Merrill et al., 2013). Extension of these studies using double labeling fluorescence or electrophysiological recording has further demonstrated that many bladder afferent neurons express receptors and ion channels that function in nociception [e.g., transient receptor potential (TRP) channels] (Skryma et al., 2011; Avelino et al., 2013). Together these studies are progressively defining the functional phenotypes of bladder afferent neurons.

DRG neurons express receptors (GFRα1–3) for three members of the glial cell line-derived neurotrophic factor (GDNF) family of ligands (GFL)—GDNF, neurturin, and artemin. Of these, only the artemin receptor (GFRα3) is associated with the peptidergic class of C-fiber neurons that is expressed by approximately half of this population (Orozco et al., 2001; Bespalov and Saarma, 2007; Ernsberger, 2008). These neurotrophic factors have powerful sensitizing actions on nociceptors and in inflamed tissue contribute to hypersensitivity and hyperalgesia (Elitt et al., 2006; Malin et al., 2006, 2011; Tanaka et al., 2011). GDNF levels in the bladder are also increased during bladder inflammation (Vizzard, 2000); to date, the levels of neurturin and artemin have not been examined here. Many sacral bladder afferent neurons express the preferred receptors for GDNF and artemin (GFRα1 and GFRα3, respectively), and these two neuron classes have distinct central projection patterns in sacral spinal cord, indicating different functions (Forrest and Keast, 2008). Following chronic bladder inflammation, the intensity of GFRα1 immunolabeling increases in the sacral dorsal horn, potentially due to structural remodeling (e.g., sprouting), increased receptor expression or increased trafficking of receptor to central terminals (Forrest and Keast, 2008). Together these results indicate these two neurotrophic factors may be involved in plasticity of sacral visceral afferent neurons following inflammation but do not identify which types of bladder afferents are likely to be most influenced by these factors.

The majority of bladder afferent neurons express estrogen receptors (ERs); many of these neurons are peptidergic and co-express TRPV1 (Bennett et al., 2003). Numerous studies have implicated estrogens in pain modulation e.g., (Robbins et al., 1990, 1992; Sanoja and Cervero, 2005; Gintzler and Liu, 2012), including direct attenuation of TRPV1 responses to capsaicin (Xu et al., 2008). In particular, hormonal status influences bladder activity, as well as the development and severity of a number of pelvic visceral pain conditions including interstitial cystitis (Shea et al., 2000; Johnson and Berkley, 2002; Robinson and Cardozo, 2003; Dmitrieva and Berkley, 2005; Warren et al., 2011). Understanding the precise targets of estrogens in bladder sensory pathways is critical to understanding their function and potentially targeting this mechanism to modulate pain.

The *first* aim of this study was to define the chemical classes of lumbar and sacral bladder sensory neurons that express receptors for two types of endogenous modulators of nociceptor sensitivity, the GFLs, and estrogens. Our strategy was to use well-described markers of key functional classes of neurons, including CGRP, TRPV1 [a marker of nociceptive C-fibers (Julius and Basbaum, 2001)] and NF200 [a marker of primary afferent neurons with myelinated axons, (Lawson et al., 1993, 1997)]. An additional outcome of this group of experiments was the first immunohistochemical characterization of myelinated bladder afferent neurons that have an important role in the micturition reflex (Sengupta and Gebhart, 1994; Yoshimura et al., 2003; Fowler et al., 2008; De Groat and Yoshimura, 2009). Our *second* aim was to identify and chemically characterize bladder sensory neurons that are most susceptible to the acutely altered environment of inflammation induced by CYP; this treatment is associated with acute but transient urothelial loss, potentially exposing underlying afferent terminals to the luminal environment (Birder, 2011; Birder et al., 2012). To identify neurons potentially undergoing structural change in response to this tissue damage, we visualized expression of the immediate early gene product, ATF-3 (activating transcription factor-3) in bladder afferent neurons after acute CYP treatment. ATF-3 can be induced in DRG following axotomy, application of noxious chemical stimuli and cellular stress (Hai et al., 1999; Tsujino et al., 2000; Averill et al., 2004; Shortland et al., 2006; Seijffers et al., 2007; Braz and Basbaum, 2010). Other studies have demonstrated that ATF-3 can also be up-regulated in models of joint inflammation and partial urethral obstruction (Xu et al., 2011). We therefore, investigated if acute bladder inflammation induces ATF-3 expression in DRGs and, if so, if this occurred in a particular chemical class.

## Materials and methods

### Animals

All procedures were approved by the Animal Care and Ethics Committees of the University of Sydney and Royal North Shore Hospital, as required by the Australian Code of Practice for the Care and Use of Animals for Scientific Purposes (National Health and Medical Research Council of Australia). Adult female Sprague-Dawley rats (6–10 weeks) were used for these experiments. They were purchased from the Animal Resources Centre (Murdoch, WA, Australia), and housed under a 12 h light-dark cycle with free access to food and water. Estrous cycle was not monitored or controlled in these experiments.

### Retrograde tracer injections and animal treatments

To identify bladder-projecting afferent neurons in DRGs, the retrograde tracer Fluorogold (FG; Fluorochrome, Englewood, CO; 4% in sterile saline; total <10 μ l) was injected into ~8 sites in the urinary bladder base using an insulin syringe with a 30 G needle, as described previously (Forrest and Keast, 2008). This was performed under isoflurane anaesthesia (3% for induction, 1.5–2% for maintenance in O_2_). Animals used only for retrograde tracing studies were perfused with fixative 7 days after surgery (see below). A second group of animals underwent retrograde tracing and 7 days later acute bladder inflammation was induced. This was performed by undergoing brief anaesthesia with isoflurane (3%) during injection with a single dose of CYP (75 mg/kg in sterile saline, i.p.) or saline (1 ml/kg, i.p.), as described previously (Corrow and Vizzard, 2007). These rats were perfused with fixative 24 h later (see below).

### Tissue preparation and immunohistochemistry

Rats were heavily anaesthetised with sodium pentobarbitone (80 mg/kg i.p.) and transcardially perfused with 0.9% saline containing 1.25% sodium nitrite and 0.036% heparin, followed by freshly made 4% paraformaldehyde in 0.1 M phosphate buffer (PB, pH 7.4). DRGs (L1, L2, L6, S1) and the bladder were removed and post-fixed overnight in the same fixative at 4°C, then washed in 0.1 M phosphate buffered saline (PBS, pH 7.2) and stored in PBS containing 0.1% sodium azide until sectioning.

DRGs were cryoprotected overnight in PBS containing 30% sucrose and cut on a cryostat into 14 μm sections. Sections were collected onto 0.1% gelatinized slides and distributed between slides so that sections stained for the same substance were sampled at least 56 μm apart. Each block contained two DRGs, each from the same spinal level, and comprising one each from a saline and CYP-treated animal. Sections were air-dried, washed in PBS, and blocked for 1–2 h in PBS containing 10% non-immune horse serum and 0.1% triton X-100.

Sections were incubated overnight in combinations of antisera, washed, and incubated for 2–3 h in appropriate host-specific secondaries (Table 1). Each of the primary antibodies has been characterized extensively in previous studies (Forrest and Keast, 2008; Kalous et al., 2009; Kiasalari et al., 2010) and showed general patterns of staining comparable to these earlier reports. To identify neurons that could potentially be influenced by GFLs, we used antibodies raised against the preferred receptors for GDNF (GFRα1), neurturin (GFRα2), and artemin (GFRα3). In this study, we utilized two different ERα antibodies, the first purchased from Affinity Bioreagents has been used previously by our laboratory (Bennett et al., 2003), and the second from Millipore that has been reported to label a different population of neurons (Kiasalari et al., 2010). Here we have distinguished these two ERα antibodies by their manufacturer, i.e., ERα (Affinity) and ERα (Millipore). The antibody from Affinity was raised against the N-terminal 21 residues of the receptor, whereas the antibody from Millipore was raised against the C-terminal 15 residues. According to information supplied by the manufacturers, specificity has been demonstrated with Western blotting, notably that neither ERα antibody cross-reacts with the most likely potential cross-reacting receptor, ERβ. We have previously determined that all ERα immunolabeled in rat DRG with the ERα (Affinity) antibody is absorbed by the antigenic peptide, including in neurons that also express ERβ; (Bennett et al., 2003) data provided by Kiasalari et al. (2010) on the ERα (Millipore) antibody, has shown patterns of staining comparable to those demonstrated by that group with in situ hybridization.

**Table 1:** **Primary and secondary antibodies used for immunohistochemistry**.

**Antigen**	**Host**	**Supplier/catalog number**	**Dilution**
**PRIMARY ANTIBODIES**			
ATF-3	Rabbit	Santa Cruz Biotechnology (Scoresby, VIC, Australia); sc-188	1:500
ED-1 (CD86)	Mouse	Millipore (Kilsyth, VIC, Australia); MAB1435	1:1000
CGRP	Goat	Millipore; 1720-9007	1:2000
CGRP	Rabbit	Sigma-Aldrich (Castle Hill, NSW, Australia); C8198	1:5000
ERα	Rabbit	Affinity Bioreagents (Scoresby, VIC, Australia); PA1-308	1:350
ERα	Rabbit	Millipore; C1355	1:5000
GFRα1	Goat	R&D Systems (Minneapolis, MN); AF560	1:400
GFRα2	Goat	R&D Systems; AF429	1:300
GFRα3	Goat	R&D Systems; AF2645	1:300
NF200	Mouse	Sigma-Aldrich; N0142	1:4000
NOS	Rabbit	Zymed Laboratories (now Invitrogen, Mulgrave, VIC, Australia); 61-7000	1:500
TRPV1	Guinea pig	Millipore; AB5566	1:5000
TRPV1	Rabbit	Neuromics (Burwood East, VIC, Australia); RA10110	1:8000
**SECONDARY ANTIBODIES**			
anti-goat Cy3	Donkey	Jackson Laboratories; 705-165-147	1:1000
anti-guinea pig FITC	Donkey	Jackson Laboratories; 706-095-148	1:100
anti-mouse AF488	Donkey	Invitrogen; A-21202	1:1000
anti-mouse Cy3	Donkey	Jackson Laboratories; 715-165-150	1:2000
anti-rabbit AF488	Donkey	Invitrogen; A21206	1:2000
anti-sheep AF488	Donkey	Invitrogen; A-11015	1:1000

Antibodies were diluted with 0.1 M hypertonic PBS (pH 7.2). All incubations were performed in a humid chamber, in the dark at room temperature. Slides were cover-slipped with carbonate-buffered glycerol (pH 8.6).

To confirm acute bladder inflammation, bladder sections (14 μm) from acute CYP-treated animals and controls were processed for ED-1, as described for DRG sections. Qualitative assessment showed a pronounced infiltration of ED-1-positive cells (likely monocytes and macrophages) in the detrusor and mucosa of acute CYP-treated animals but not in the saline group (not shown).

### Quantitation of neuronal populations

DRGs were viewed under an Olympus BX51 fluorescence microscope (Olympus Australia, Melbourne, Australia) and counts made while viewing directly from the microscope. To characterize bladder-projecting neurons, a minimum of 120 nucleated profiles of FG-labeled neurons were counted from five sections from each ganglion. Ganglia were classified as “lumbar” if from spinal levels L1 or L2 and “sacral” if from L6 and S1. These neurons were classified on the basis of their immunoreactivity (IR) for GFRs, TRPV1, CGRP, ERα (both antibodies), NF200 and NOS. The percentage of FG neurons expressing each marker or co-marker was averaged for each DRG in each rat. The arcsine-transformed data from sacral and lumbar DRGs was compared using a paired *t*-test. The number of ATF-3 positive FG-labeled neurons containing CGRP, NOS, and the GFRs following acute CYP treatment or saline were compared by performing Tukey's multiple comparison *post hoc* test on arcsine transformed data. This was used to detect an effect of CYP treatment on the proportion of FG-labeled neurons expressing each of these markers and a comparison between sacral and lumbar DRGs. In past studies, sacral DRGs have been more thoroughly characterized so will be the primary point of reference and data on these neurons will be provided first in each of the histograms and in the text. All results are provided as the mean ± s.e.m. and *P* < 0.05 was regarded as statistically significant.

### Figure production

Eight-bit monochrome images were captured with an RT-SPOT camera (Diagnostic Instruments, Sterling Heights, MI) and digitized using Image Pro Plus (Media Cybernetics, Silver Spring, MD). For figure production, only minor adjustments were made to brightness and contrast in Adobe Photoshop (San Jose, CA) to best represent the staining as viewed directly under the microscope. Colorized images were produced by pasting monochrome images into the appropriate color channel of a 24-bit RGB file in Photoshop; these images were collated and labeled using InDesign (Adobe Creative Suite 5). FluoroGold labeling was colorized blue instead of its native color to facilitate viewing of merged images.

## Results

### GFRα1 and GFRα3 were expressed in many TRPV1-IR bladder-projecting neurons

FG-labeled neurons were identified in all of the ganglia and we first quantified how many of these neurons were immunoreactive for each of CGRP, the GFRs, and TRPV1. TRPV1 is a marker of polymodal nociceptor neurons that is expressed by the majority of bladder afferent neurons (Bennett et al., 2003). Previous studies of DRGs at other spinal levels have shown that the majority of CGRP-IR neurons express TRPV1 (Kiasalari et al., 2010) and that GFRα3-IR is expressed in a sub-population of CGRP-IR neurons (Orozco et al., 2001; Kalous et al., 2009). From these previous studies we also predicted that GFRα1- and GFRα2-IR would be found in the non-peptidergic population (Bennett et al., 1998; Kalous et al., 2007, 2009).

**Figure 1:**
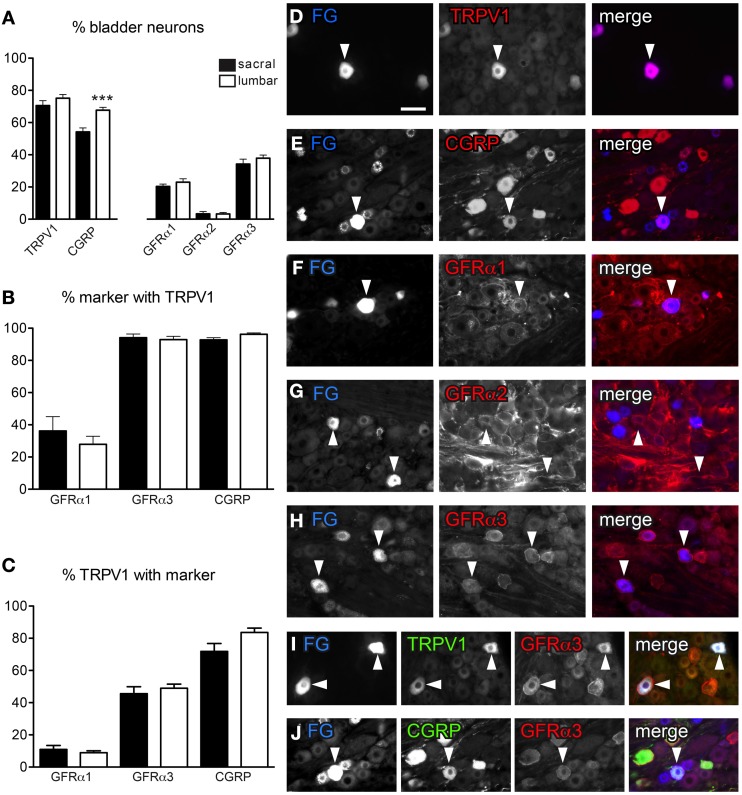
**Distribution of TRPV1-, CGRP-, and GFRα-immunoreactivity (IR) in bladder sensory neurons. (A)** The proportion of bladder sensory neurons containing TRPV1-, CGRP-, and GFR-(IR) at each spinal level. Bladder-projecting neurons were identified with the retrograde tracer, FluoroGold (FG). A higher proportion of FG-labeled neurons contain CGRP-IR in lumbar than sacral DRGs (^***^P < 0.001). (**B)** The proportion of GFRα1-, GFRα3-, and CGRP-IR bladder sensory neurons co-expressing TRPV1-IR. **(C)** The proportion of TRPV1-IR bladder sensory neurons co-expressing GFRα1-, GFRα3-, or CGRP-IR. **(D–J)**: Each row of micrographs comprises images from the same field, showing immunostaining for one or more markers in retrogradely-labeled bladder sensory neurons. The first panel shows the tracer dye, FluoroGold (FG), the second panel shows an immunolabel and the last panel provides a merged, colorized image. The color of each label in the merged image is indicated by the color of the text in the relevant monochrome image. **(D–H)** Example of FG neurons expressing TRPV1- **(D)**, CGRP- **(E)**, GFRα1- **(F)**, and GFRα3-IR **(H)**. Very few FG neurons were labeled for GFRα2 **(G)**. **(I, J)** Co-expression of GFRα3 with TRPV1 **(I)** and CGRP **(J)** in FG-labeled bladder-projecting neurons. Histograms show the mean ± s.e.m. (*n* = 4), analyzed by a paired *t*-test on arcsine-transformed data. Scale bar in panel **(D)** represents 50 μm and applies to all micrographs. Arrowheads indicate examples of retrogradely labeled neurons and their relative location in matching panels.

In sacral DRG, 70.6 ± 3.1% of FG-labeled neurons contained TRPV1-IR and 54.2 ± 2.5% contained CGRP-IR (*n* = 4, Figures 1A,D,E). Expression of each GFR in retrogradely labeled neurons varied with the type of receptor, with 20.4 ± 1.4% GFRα1-IR, <2% GFRα2-IR and 34.3 ± 3.0% GFRα3-IR (*n* = 4, Figures 1A,F–H). Quantification of FG-labeled neurons in lumbar DRG found broadly similar patterns of labeling (*n* = 4; Figure 1A), although consistent with previous publications (Bennett et al., 2003), we found CGRP-IR neurons more prevalent in lumbar than sacral DRGs (67.7 ± 1.8% lumbar vs. 54.2 ± 2.5% sacral, *n* = 4, *P* < 0.001, Figure 1A).

We next investigated the co-expression of GFRα1-, GFRα3-, and CGRP-IR with TRPV1-IR in bladder afferent neurons (Figures 1B,C,I,J). This was not performed with GFRα2 as it was expressed by so few FG-positive neurons. At both spinal levels, approximately one third of GFRα1-IR bladder afferent neurons contained TRPV1-IR (Figure 1B), but these comprised only ~10% of all TRPV1-IR bladder afferent neurons (Figure 1C). Almost all GFRα3- and CGRP-IR neurons contained TRPV1-IR (Figures 1B,I,J). Conversely, approximately half of all TRPV1-IR bladder neurons contained GFRα3-IR (Figures 1C,I) and 70–80% contained CGRP-IR (Figures 1C,J). In both sacral and lumbar DRGs, almost all GFRα3-IR bladder neurons contained CGRP-IR (sacral, 94.3 ± 2.1%; lumbar, 96.7 ± 1.4%; *n* = 4) and the majority of CGRP-IR bladder neurons were GFRα3-IR (sacral, 70.1 ± 2.8%; lumbar, 61.2 ± 2.3%, *n* = 4).

### ERα was expressed in functionally diverse populations of bladder sensory neurons

**Figure 2:**
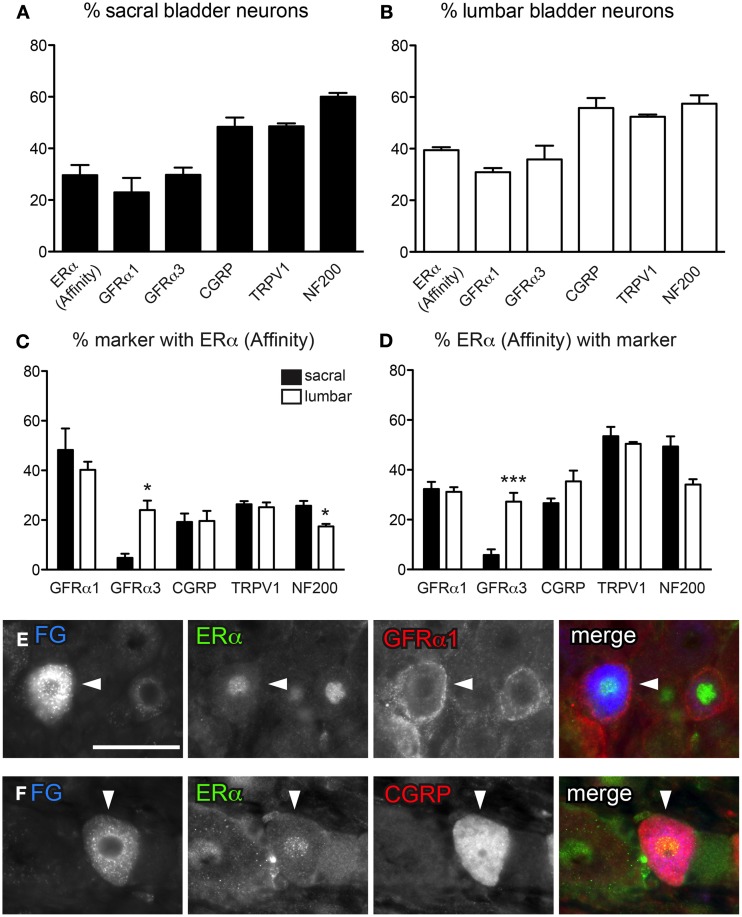
**Distribution of ERα and GFRs in bladder sensory neurons**. This figure illustrates immunostaining for ERα using the antibody purchased from Affinity Bioreagents. **(A, B)** The proportion of sacral **(A)** and lumbar **(B)** bladder-projecting neurons containing ERα-, GFRα1, GFRα3-, CGRP-, TRPV1-, and NF200-immunoreactivity (IR). **(C)** The proportion of GFRα1-, GFRα3-, CGRP-, TRPV1-, and NF200-IR bladder sensory neurons containing ERα-IR. A higher proportion of GFRα3-IR neurons contained ERα-IR in lumbar vs. sacral DRGs (^*^P = 0.01), whereas a higher proportion of NF200-IR neurons contained ERα-IR in sacral vs. lumbar DRGS (^*^P = 0.03). **(D)** The proportion of ERα-IR bladder sensory neurons containing each marker. A higher proportion of ERα-IR neurons contained GFRα3-IR in lumbar vs. sacral DRGs (^***^P < 0.001). Data represents the mean ± s.e.m. (*n* = 5), analyzed by a paired *t*-test on arcsine-transformed data. **(E)** Co-expression of ERα- and GFRα1-IR in a FluoroGold (FG)-labeled neuron. **(F)** Co-expression of ERα- and CGRP-IR in a FG-labeled neuron. Images are shown as monochrome image sets and the merged colorized image. The color of each label in the merged image is indicated by the color of the text in the relevant monochrome image. Scale bar in panel **(E)** represents 50 μm and applies to all images. Arrowheads indicate examples of retrogradely labeled neurons and their relative location in matching panels.

DRG sections immunostained with either ERα antibody labeled the nucleus of a subpopulation of neurons; neurons with a nucleus brighter than the cytoplasm were regarded as ERα-IR. In some neurons stained with the Affinity antibody, in addition to ERα-positive nuclei there was weak cytoplasmic staining. Assessment of FG-labeled neurons labeled with this antibody showed that in sacral and lumbar DRGs, 29.6 ± 3.9% and 39.4 ± 1.2%, respectively, were immunoreactive for ERα (*n* = 5, Figures 2A,B). In these double-stained sections, we found a similar pattern of expression for GFRs and TRPV1 (Figure 2B) as described above. However, in this component of the study we also co-stained for the myelination marker, NF200, and found that ~60% of sacral and lumbar FG-labeled neurons were immunoreactive for NF200 (*n* = 5, Figures 2A,B).

Using the antibody from Affinity, ERα-IR neurons were then categorized according to their co-expression with GFRα1, GFRα3, CGRP, TRPV1, and NF200 (Figures 2C–F). Approximately 40% of GFRα1-IR FG-labeled neurons in sacral and lumbar DRGs contained ERα-IR, and these comprised ~30% of all ERα-IR neurons. However, a difference between bladder sensory neurons in the sacral and lumbar DRG was revealed with GFRα3 co-labeling—very few sacral GFRα3-IR neurons were ERα-IR, compared to ~20% of these neurons being ERα-IR in lumbar DRG (*n* = 5, *P* = 0.01). This difference was also reflected in the proportion of ERα-IR neurons containing GFRα3-IR (5.8 ± 2.3% sacral vs. 27.3 ± 3.6% lumbar; *n* = 5, *P* < 0.001). Using this same antibody purchased from Affinity, the co-expression of ERα with TRPV1 and CGRP has previously been reported in bladder sensory neurons (Bennett et al., 2003). The current study using this antibody showed similar results, with 20–30% of all CGRP- and TRPV1-IR bladder sensory neurons in lumbar and sacral DRG showing ERα-IR. Conversely, ~30% of ERα-IR bladder sensory neurons contained CGRP-IR and half contained TRPV1-IR.

The expression of ERα has not previously been compared between myelinated and unmyelinated bladder sensory neurons. Using the same ERα antibody as above (from Affinity) and immunoreactivity for NF200 to indicate myelination, we found that in sacral DRGs only ~25% of myelinated bladder neurons were labeled for ERα-IR; even fewer (~15%) of this population expressed ERα in lumbar DRGs (Figures 2C,D; *P* = 0.03, *n* = 5). When viewing the co-expression from the perspective of the ERα-positive population of bladder sensory neurons, in sacral DRG around half of these were myelinated and potentially fewer of these were myelinated in lumbar DRG (although this did not quite reach statistical significance; *P* = 0.05, *n* = 5).

The ERα antibody obtained from Millipore produced a similar pattern of IR in the nucleus of DRG neurons as that obtained by Affinity, however, the Millipore antibody labeled fewer bladder afferent neurons. As a proportion of FG-labeled neurons, the Millipore antibody labeled 17.9 ± 1.8% (sacral) and 13.5 ± 1.5% (lumbar) of FG neurons, whereas the Affinity antibody labeled 29.6 ± 3.9% (sacral) and 39.4 ± 1.2% (lumbar) of bladder neurons (all *n* = 5). We then wished to determine if this decrease was due to a failure of the Millipore antibody to stain a particular population of neurons labeled as ERα by the Affinity antibody; if so, this could reveal a difference in specificity for a form of the receptor that is distributed differently between neuronal classes.

These additional studies showed that, in contrast to the Affinity antibody, the Millipore antibody failed to label ERα in peptidergic bladder sensory neurons. It was rare to find bladder sensory neurons that co-expressed ERα with GFRα3- or CGRP-IR in either lumbar or sacral DRG. However, using the Millipore antibody, co-expression with GFRα1 in sacral bladder sensory neurons was observed to a similar degree as with the antibody purchased from Affinity. Specifically, at this spinal level ~25% GFRα1-IR bladder sensory neurons contained ERα-IR, and these comprised approximately half of all ERα-IR neurons. Lumbar bladder sensory neurons showed less expression of ERα using the Millipore antibody, with only ~5% of GFRα1-IR neurons containing ERα-IR, and these comprising ~10% of all ERα-IR neurons. Further examination of the labeling obtained with these two antibodies was performed in the context of myelination status (see below).

### Characterization of myelinated bladder afferent neurons

**Figure 3:**
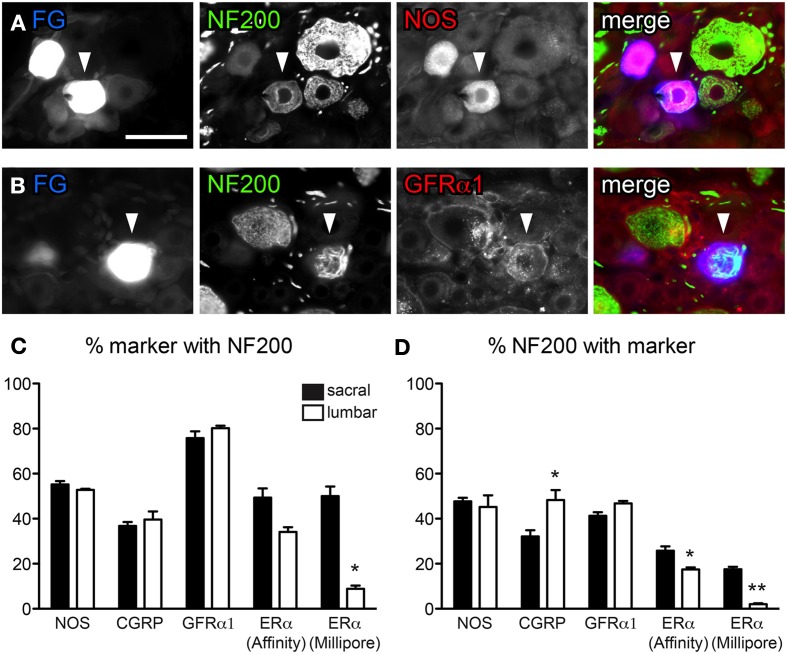
**Distribution of NF200-immunoreactivity (IR) in bladder sensory neurons**. Data shown here compares two antibodies raised against ERα, purchased from either Affinity Bioreagents or Millipore. **(A, B)** Co-expression of NF200 with NOS **(A)** and GFRα1 **(B)** in bladder-projecting neurons retrogradely labeled with FluoroGold (FG). The neurons in panel **(B)** are from the same field of view as Figure 1F. Images are shown as monochrome image sets and the merged image. The color of each label in the merged image is indicated by the color of the text in the relevant monochrome image. Scale bar in panel **(A)** represents 50 μm and applies to all images. **(C)** The proportion of NOS-, CGRP-, GFRα1-, and ERα-IR bladder-projecting neurons containing NF200-IR. Using the Millipore antibody, a higher proportion of ERα-IR neurons contain NF200-IR in sacral than lumbar DRGs (^*^P = 0.02). **(D)** The proportion of NF200-IR bladder afferent neurons containing each marker. A higher proportion of NF200-IR neurons contain CGRP-IR in lumbar than sacral DRGs (^*^P = 0.02). Using either ERα antibody, a higher proportion of NF200-IR bladder afferent neurons contain ERα in sacral compared to lumbar DRGs (^*^P = 0.03 for ERα [Affinity], ^**^P < 0.01 for ERα [Millipore]). Data represents the mean ± s.e.m. (*n* = 5), analyzed by a paired *t*-test on arcsine-transformed data.

Previous studies have reported that just over half of all bladder afferent neurons express the myelination marker, NF200-IR (Hayashi et al., 2009), but its distribution in different chemical classes is unknown. NF200 antibody labeled many DRG neurons with varying intensity, with the largest diameter labeled neurons having the brightest staining (Figures 3A,B). Many NF200-IR axons were also observed running through the DRG. Consistent with previous reports (Hayashi et al., 2009), we found just over half of all sacral and lumbar bladder-projecting neurons contained NF200-IR (54.8 ± 1.6% sacral; 57.3 ± 1.4% lumbar, *n* = 3).

We then performed double-labeling studies to determine if myelinated bladder sensory neurons express nitric oxide synthase (NOS), CGRP, GFRα1, or ERα (using both sources of ERα antisera). Assessment of FG-labeled neurons in sacral and lumbar DRG for the other neuronal classes (CGRP, GFRα1, and ERα) showed that each class comprised both myelinated (NF200-IR) and unmyelinated (NF200-negative) neurons, however, their relative distributions between these populations differed (Figures 3C,D). When all populations of myelinated neurons were analyzed separately they totaled more than 100% (Figure 3D), as would be expected given that many neurons will actually express more than one of these substances (e.g., ERα with CGRP).

NOS activity has been linked to plasticity of visceral afferent pathways and is expressed in some bladder afferent neurons (Vizzard et al., 1995a,b, 1996; Callsen-Cencic and Mense, 1997). NOS-IR was of variable labeling intensity and localized to medium sized neurons. About half of all bladder neurons contained NOS-IR (sacral, 45.9 ± 1.5%; lumbar, 46.6 ± 3.1%; Figure 3A, *n* = 3). This is a much higher basal expression of NOS than reported previously (Vizzard et al., 1995a,b, 1996; Callsen-Cencic and Mense, 1997) and may reflect differences between antisera or in the level of labeling intensity that was regarded as positive. Just over half of all NOS-IR bladder neurons contained NF200-IR and, in turn, these comprised almost half of all NF200-IR neurons (Figures 3A,C,D); this did not vary between lumbar and sacral DRG.

At each spinal level, ~40% of CGRP-IR bladder neurons were NF200-IR (Figure 3C), however, more of these NF200-IR neurons expressed CGRP in the lumbar than the sacral DRG (48.3 ± 4.4% lumbar vs. 32.1 ± 2.0% sacral; Figure 3D, *P* = 0.02, *n* = 3). Approximately 80% of GFRα1-IR bladder neurons contained NF200-IR and these comprised ~40% of all NF200-IR bladder neurons (Figures 3B–D); this did not vary between lumbar and sacral DRG.

Expression of ERα relative to myelination status was more complex, showing differences between spinal levels and between the two ERα antibodies (Figures 3C,D). Using the antibody from Affinity, the proportion of ERα-IR bladder neurons containing NF200-IR may have been higher in sacral than lumbar DRGs; this was close to reaching statistical significance (*n* = 5, *P* = 0.05). In line with this observation, a higher proportion of NF200-IR bladder neurons contained ERα-IR in sacral than lumbar DRGs (*n* = 5, *P* = 0.03). Although the ERα antibody obtained from Millipore labeled a smaller proportion of neurons than the Affinity antibody, similar patterns of co-expression were observed relative to NF200. That is, a higher proportion of ERα-IR bladder neurons contained NF200-IR in sacral DRGs compared to lumbar DRGs (*n* = 5, *P* = 0.02). A higher proportion of NF200-IR neurons also contained ERα-IR in sacral than lumbar DRG (*P* = 0.02, *n* = 5).

### Acute bladder inflammation induced ATF-3 activation and increased NOS expression in sacral bladder afferent neurons

**Figure 4:**
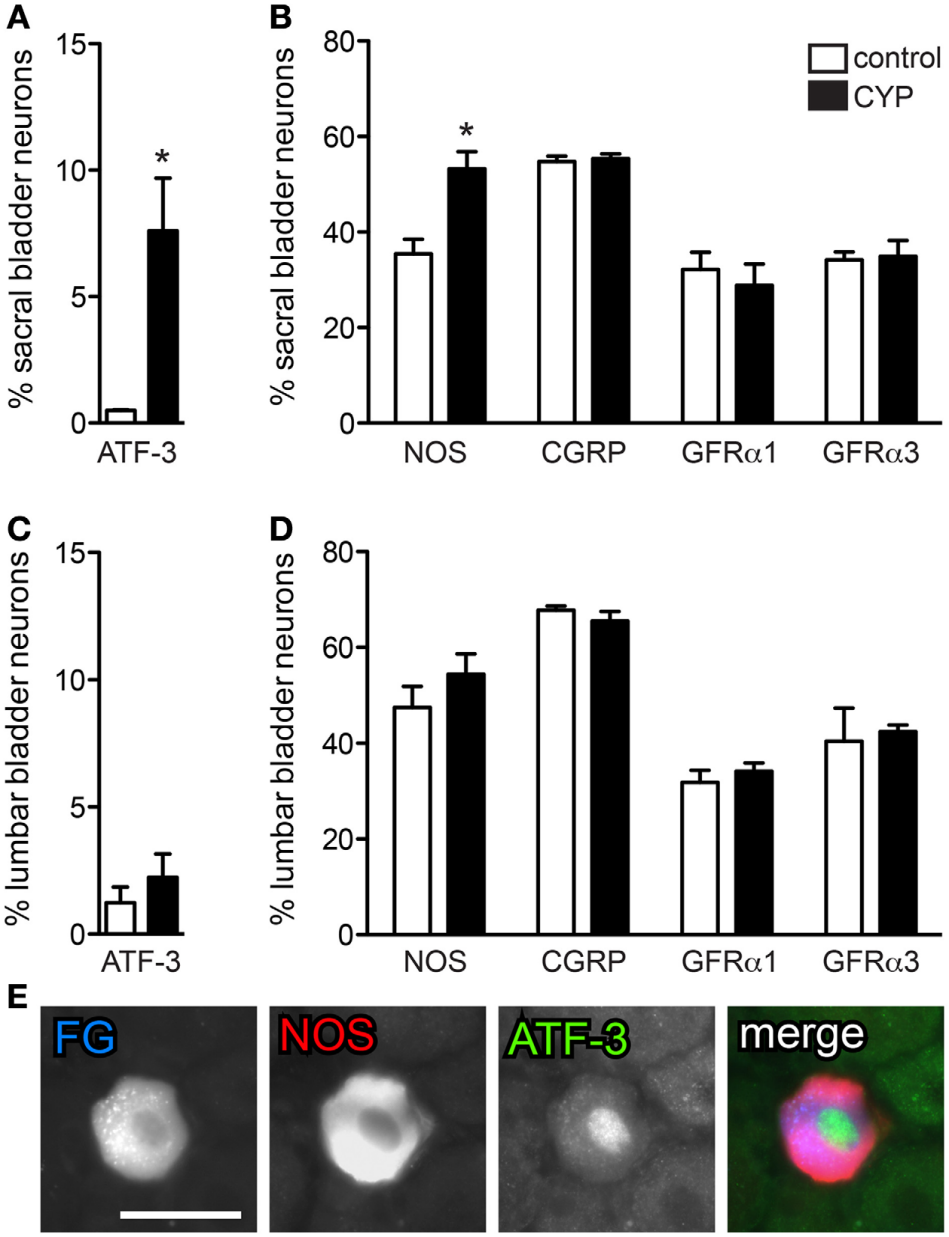
**Acute bladder inflammation induced ATF-3 and increased NOS-IR in sacral bladder sensory neurons. (A)** Acute cyclophosphamide (CYP) treatment caused a small, but significant induction of ATF-3 in sacral bladder sensory neurons (^*^P = 0.03). **(B)** In sacral DRG, acute CYP treatment increased the proportion of FG-labeled neurons containing NOS-IR (^*^P = 0.01) but did not affect the prevalence of CGRP-, GFRα1-, or GFRα3-IR bladder sensory neurons. **(C)** In lumbar DRG, CYP treatment did not affect the proportion of lumbar bladder sensory neurons containing ATF-3. **(D)** Acute CYP treatment did not affect the proportion of lumbar bladder sensory neurons with NOS-, CGRP-, GFRα1-, or GFRα3-IR. **(E)** Approximately 80% of ATF-3-IR nuclei were found in NOS-IR neurons. Data represents the mean ± s.e.m. (*n* = 3), analyzed by a paired *t*-test on arcsine-transformed data. Images are shown as monochrome image pairs and the merged image. The color of each label in the merged image is indicated by the color of the text in the relevant monochrome image. Scale bar in the first panel (left) represents 30 μm and applies to all images.

FG-labeled neurons were assessed for presence of the transcription factor, ATF-3. Following acute CYP treatment there was a small, but significant induction of ATF-3 in bladder neurons in sacral DRG (Figure 4A; *P* = 0.03, *n* = 3). ATF-3 neurons were rarely seen in DRG from saline treated animals. We also observed an increase in the number of neurons expressing NOS-IR (Figure 4B; *P* = 0.01; *n* = 3) compared to control animals, but there was no effect of CYP on the proportion of bladder sensory neurons labeled for CGRP, GFRα1, or GFRα3 (Figure 4B). Co-expression studies revealed that ~80% of ATF-3 nuclei were found in NOS-IR neurons (Figure 4E). In contrast to sacral bladder afferent neurons, acute CYP treatment had no effect on the expression of ATF-3, NOS-, CGRP-, GFRα1-, or GFRα3-IR in lumbar FG-labeled neurons (Figures 4C,D).

## Discussion

This is the first study to identify the populations of bladder sensory neurons in lumbar and sacral DRG that express receptors for the GDNF family of neurotrophic factors and estrogens, and are potentially influenced by these endogenous modulators of nociception. The results demonstrated that diverse but not identical groups of neurons express these receptors and some bladder sensory neurons express both families of receptors. Lumbar and sacral sensory neurons showed some distinct differences in their labeling profiles. We also identified a small population of bladder sensory neurons in sacral DRG that upregulate the injury-related transcription factor, ATF-3, following acute systemic administration of CYP. We propose that these are the neurons most likely to be damaged and undergoing structural remodeling during inflammation. This remodeling may drive the development of abnormal behavior (spontaneous activity, sensitization) in the chronic setting.

It has previously been shown that populations of bladder sensory neurons express receptors for GDNF and artemin (i.e., GFRα1 and GFRα3, respectively) (Forrest and Keast, 2008). The central projections of sacral sensory neurons expressing these receptors terminate in different locations within the dorsal horn of the spinal cord, illustrating their distinct roles within micturition (and potentially nociceptive) circuitry (Forrest and Keast, 2008). By performing double-labeling immunofluorescence, we have shown that, similar to DRGs from other spinal levels, these two receptors are associated with different populations of neurons—GFRα1 with non-peptidergic and GFRα3 with peptidergic neurons (Bennett et al., 1998, 2000; Orozco et al., 2001). Therefore, if levels of GDNF or artemin increase in disease states such as chronic inflammation, they would be predicted to influence different aspects of bladder function. Moreover, GFRα3-positive are peptidergic so also express the nerve growth factor receptor, trkA (Averill et al., 1995), increasing the complexity of possible mechanisms for their peripheral sensitization (Pezet and Mcmahon, 2006).

Both groups of bladder sensory neurons (GFRα1- and GFRα3-IR) include neurons that could be considered nociceptive by their expression of TRPV1. These comprise a minority of the GFRα1-IR bladder afferents and the majority of GFRα3-IR bladder afferents. None of the bladder afferents label for the receptor of the related neurotrophic factor, neurturin, although many pelvic autonomic neurons (including bladder motor neurons) express this receptor (Laurikainen et al., 2000; Wanigasekara et al., 2004; Wanigasekara and Keast, 2005). It is not known if GFRα2 is expressed by sensory neurons innervating other pelvic viscera, although there is functional evidence for its presence in other populations of afferents (Stucky et al., 2002; Lindfors et al., 2006; Malin et al., 2006). TRPV1 is generally considered a marker of polymodal nociceptors (Julius and Basbaum, 2001) but many TRPV1-IR visceral afferent neurons are not exclusively involved in nociception and are likely to play a role in normal autonomic regulation, including the micturition reflex (De Groat and Yoshimura, 2001, 2009; Robinson and Gebhart, 2008). For example, intravesical injection of capsaicin into the bladder decreases the micturition threshold and induces bladder contraction (Maggi, 1992; Dmitrieva et al., 1997). Some bladder neurons develop a nociceptive function under inflammatory conditions (Bjorling et al., 2003; Nazif et al., 2007). At other spinal levels, GDNF and artemin contribute to nociceptor sensitization and hyperalgesia (Elitt et al., 2006; Malin et al., 2006, 2011). Artemin is also likely to contribute to the development and maintenance of colorectal hypersensitivity (Tanaka et al., 2011). GDNF levels in the bladder are increased during bladder inflammation (Vizzard, 2000) but the expression of artemin by the rat bladder is unknown. Together these results indicate that GDNF and artemin may be involved in the sensitization and plasticity of pelvic visceral pathways.

Many bladder afferent neurons express ERs (Bennett et al., 2003) and various actions of estrogens have been reported on these neurons. Estrogens have complex actions on bladder afferent neurons where they modulate two signaling pathways involved in neural plasticity and nociceptor sensitization, p38 and extracellular signal-related (ERK) mitogen-activated protein (MAP) kinase (Cheng and Keast, 2009). Another study (Xu et al., 2008) has shown that estradiol acts directly on DRG neurons to reduce TRPV1 activation by capsaicin and suggested that pain could be influenced by modulation of ERs in DRG neurons. Here we used two different antibodies, one of which (Affinity Bioreagents) has been used previously by our laboratory to identify ERα in bladder sensory neurons in rats (Bennett et al., 2003), and a second from Millipore that has recently been described in a study of perineal afferents (Kiasalari et al., 2010). It has been demonstrated previously that many peptidergic and TRPV1-IR bladder sensory neurons express ERs (Bennett et al., 2003). In the current work using the Affinity antibody, we showed that these receptors were also expressed by some non-peptidergic, GFRα1-IR neurons and that the majority of ERα-IR neurons are unmyelinated. These detailed analyses revealed a clear difference between bladder sensory neurons at different spinal levels, with ERα only expressed by GFRα 3-IR bladder neurons in the lumbar but not sacral cord. It will be interesting to determine how estrogen exposure influences the function of these different classes of bladder sensory neurons.

Our use of two different ERα antibodies raises questions about the nature of the antigen that is being revealed. We found a clear overlap in the population of bladder sensory neurons identified by each antibody, but the reagent from Millipore labeled many fewer neurons and specifically failed to label the peptidergic class (Kiasalari et al., 2010). It is possible that a particular isoform of the receptor is not expressed by these neurons. Other possible explanations for this discrepancy suggested by a previous study in rats (Kiasalari et al., 2010) include: the bladder afferents may have a distinct ER phenotype compared with perineal afferents analyzed in the previous study; the earlier study used male Wistar rats whereas we used female Sprague-Dawley rats; we used direct labeling using fluorophore conjugated secondary antibodies whereas the previous study used tyramide amplification in conjunction with the Millipore antibody.

Our immunohistochemical approach also allowed us to distinguish between myelinated and unmyelinated neurons. Consistent with previous studies (Yoshimura et al., 1998; Hayashi et al., 2009; Russo et al., 2013), we found that over half of all bladder afferent neurons contained NF200-IR. We predict that these are the Aδ mechanosensitive neurons with thinly myelinated axons (Lawson et al., 1993). These neurons are likely to respond to innocuous stimuli such as normal levels of bladder distension. This is the first study to define which chemical classes of bladder afferent neurons are myelinated. Our results indicate that most of the neuronal types examined in this study comprise a mixture of myelinated and unmyelinated afferents. The prevalence of myelinated neurons in each group was similar in lumbar and sacral bladder afferents, with the exception of ERα-IR neurons, where a higher proportion of these were myelinated in sacral than lumbar neurons.

In this study, we identified a number of immunohistochemical differences between lumbar and sacral bladder afferents. It would be very interesting to determine how these relate to functional differences. Lumbar and sacral components of bladder sensory afferents have been previously been distinguished in a number of ways. Most obviously, sacral but not lumbar afferents are required for the micturition reflex, and undergo major changes during inflammation that are thought to contribute to bladder symptoms and pain (Yoshimura et al., 2002; Fowler et al., 2008; De Groat and Yoshimura, 2009). However, lumbar bladder afferents also become sensitized with inflammation (Mitsui et al., 2001). Moreover, it has been demonstrated in the mouse that lumbar and sacral bladder afferents terminate in different tissues within the bladder and can be activated with different stimuli (Xu and Gebhart, 2008). A similar body of information does not yet exist for the rat bladder, but it would be interesting to define the sources of termination within the bladder wall of each neuronal population studied here. At present there are no unique markers for these spatially distinct groups of axons.

In the current study, we utilized ATF-3 expression to identify populations of bladder afferent neurons that were likely to be injured and undergoing structural remodeling during acute bladder inflammation. These were observed only in sacral DRG, where they comprised a minor class (<10%) of bladder afferent neurons. The absence of ATF-3 from lumbar DRG may indicate that these neurons do not change in response to CYP or that their terminals are more distant from the remodeling urothelium. We chose ATF-3 for this study on the basis of a previous study that observed ATF-3 induction in sensory neurons following injection of noxious irritants into the hindpaw (Braz and Basbaum, 2010). Our result is also consistent with reports of ATF-3 induction in lumbar DRGs by mono-iodoacetate-induced osteoarthritis (Ivanavicius et al., 2007), a model of joint in inflammation that produces concurrent axonal damage required to drive ATF-3 induction. In our study, a possible explanation for the ATF-3 induction is that CYP metabolism to acrolein causes a rapid destruction of the urothelium, allowing toxic substances in urine to impact on the activity and structure of suburothelial axons. These axons could potentially be sensitized and contribute to altered reflex behaviors during inflammation. However, it should be noted that CYP has additional properties and systemic effects (e.g., immunosuppression) that were not examined in this study and could contribute to our results. Nevertheless, the acuteness of the ATF-3 upregulation and its typical association with neuronal injury, provide strong support for involvement of a bladder-related mechanism, such as urothelial damage.

We also found that acute CYP induced an increase in NOS expression in some sacral (but not lumbar) bladder afferents. This raises the possibility that NOS upregulation is occurring in the ATF-3-IR neurons. Rapid up-regulation of NOS in DRGs has been reported previously following CYP (Vizzard et al., 1996) or intra-vesical mustard oil (Callsen-Cencic and Mense, 1997). Nitrergic activity in the spinal cord has been implicated in bladder hyperactivity following acute inflammation (Kakizaki and De Groat, 1996), but peripheral actions of increased nitric oxide synthesis should also be considered. Our results are also consistent with an increase of calcium-dependent NOS activity in spinal cord in association with CYP-induced bladder hyperreflexia (Lagos and Ballejo, 2004). We did not see an acute effect of CYP treatment on expression of other neuronal markers (CGRP, GFRs) but it should be recognized that by assessing only the proportion of neurons expressing a certain substance, we may fail to see smaller changes in expression. Moreover, we did not assess neuronal size, which could have potentially been altered by inflammation and, if changing, confound our sampling of neurons for quantitation.

In conclusion, we have defined for the first time the bladder sensory neurons that express receptors for GDNF, artemin, and estrogens. We have also provided a detailed characterization of myelinated bladder afferents. The complexity and heterogeneity of bladder sensory neurons is reflected at both lumbar and sacral levels but not identical in each. Our study has also identified a sub-population of NOS-IR sacral sensory neurons that are likely to be undergoing structural remodeling during acute inflammation of the bladder. Together these results contribute to increased understanding of the neurons that are known to be involved in pain modulation and hyperreflexia during inflammation.

## Author contributions

Shelley L. Forrest conducted all of the experimental work and participated in drafting the manuscript. Peregrine B. Osborne and Janet R. Keast conceived the study, participated in data analysis, and took primary responsibility for drafting the manuscript.

### Conflict of interest statement

The authors declare that the research was conducted in the absence of any commercial or financial relationships that could be construed as a potential conflict of interest.
